# Round the Hospitals

**Published:** 1917-06-23

**Authors:** 


### ROUND THE HOSPITALS.

The Nation's Fund for Nurses has been launched by a contribution to the College of Nursing of £500 from the British Women's Hospital Committee, being part of the proceeds of the season's entertainments organised by this Committee. The acceptance of votes for women by an overwhelming majority in the House of Commons, and the continuous and triumphant progress of the College of Nursing, point to the probability that at least two and possibly three noughts may be added to the £500 which at present represents the yield for the College from the British Women's Hospital Committee. We have had an opportunity of seeing and considering in detail the figures which demonstrate the development and progress of the College of Nursing. The growth of its Register since the beginning of the year has been as steady as it is remarkable. The actual figures demonstrate the wisdom exhibited by an ever-increasing number of the more thoughtful members of the British nursing profession, who follow the highest standards and believe in continuous personal service. Thousands of them have or are preparing to join the College Register and to co-operate to the utmost possible extent in the development of the College. Its speedy triumph in defence of the best interests of trained nurses throughout the Nation and Empire being now assured.

On Thursday, June 14, the superintendents and sisters of the Royal Infirmary, Edinburgh, were presented with the new badge of the training-school. The badge is the gift of a former manager, who herself made the presentation. It is in the form of an indented oval brooch, 1¼ inches high, in silver gilt and enamel. The central design, that of a pelican feeding her young, taken from the crest of the hospital, is in bas-relief, and round the edge is a dark-blue enamel ribbon, on which is inscribed in gold "Royal Infirmary, Edinburgh, School of Nursing." The name of each recipient appears on her brooch, together with her number on the hospital register. This badge will be supplied to former certificated nurses of Edinburgh Royal Infirmary who obtain the authority of the lady superintendent to purchase it. It costs 6s. 6d., and has been designed and executed by Messrs. Hamilton and Inches, goldsmiths, Edinburgh. In future it will be issued to each nurse with her certificate of training.

The annual meeting of the Royal British Nurses' Association on June 15 was clouded by the recent and sudden death of the vice-chairman, Dr. Bezly Thorne, who was known to and beloved by a large number of members. To many of these the first announcement of his death came when H.R.H. Princess Christian, who presided, proposed a resolution placing on record the high appreciation of the President and members of the Royal British Nurses' Association of the valuable services so cheerfully rendered to the Corporation by Dr. Bezly Thorne and their sincere regret at the loss of a friend whose unfailing courtesy and kindly personality gained the respect and esteem of every one of them. They desired to convey to the family of Dr. Thorne the expression of their warm sympathy. The meeting was largely attended by members of the nursing profession, a notable tribute in these busy days to the widespread influence of the Royal British Nurses' Association. The accounts of the General Fund of the Corporation show a healthy tendency, the income exceeding the expenditure by £44.

An English trained nurse, who has had considerable administrative experience, writes from a large hospital in Belgium, where she is in charge of a floor, in high praise of the food, cooking, and serving in that institution. She ventures the bold statement that it is a pity in her opinion that the matrons and housekeepers of English hospitals cannot go out to that particular hospital to serve for a while under the lady who has charge of the catering department. That there is much to be learnt in this respect from our neighbours across the Channel is undoubted. In the articles which are appearing in our pages, "Food in War-Time" stress has been laid on French methods of cooking both meat and vegetables, and the use of well-made soup, as a food of high value. A lack of variety in the feeding of the nursing and domestic staffs of our hospitals and institutions is too often apparent, and the recent substitution by the Metropolitan Asylums Board authorities of a revised menu, introducing more homely dishes, such as haricot ox-tails, tripe and onions, stews, and so forth, in place of the eternal roast beef and roast mutton, has been heartily welcomed.

